# Epigenetic Skeletal Muscle Memory: The Impact of Physical Activity on Aging and Post-Injury Regeneration

**DOI:** 10.3390/genes17080964

**Published:** 2026-08-17

**Authors:** Antoni Godlewski, Marcin Wróblewski, Julia Kuk, Magdalena Moritz, Filip Dobrak, Renata Kołodziejska, Alina Woźniak

**Affiliations:** 1 Student Scientific Club of Biochemistry and Bioorganic Chemistry, Department of Medical Biology and Biochemistry, Faculty of Medicine, Ludwik Rydygier Collegium Medicum in Bydgoszcz, Nicolaus Copernicus University in Toruń, 24 Karłowicza St., 85-092 Bydgoszcz, Poland; 319905@stud.umk.pl (A.G.); 319910@stud.umk.pl (J.K.); 319915@stud.umk.pl (M.M.); 319903@stud.umk.pl (F.D.); 2 Department of Medical Biology and Biochemistry, Faculty of Medicine, Ludwik Rydygier Collegium Medicum in Bydgoszcz, Nicolaus Copernicus University in Toruń, 24 Karłowicza St., 85-092 Bydgoszcz, Poland; renatak@cm002eumk.pl

**Keywords:** skeletal muscle, muscle regeneration, epigenetic memory, muscle memory, sarcopenia, aging, physical exercise

## Abstract

Skeletal muscle retains adaptive information from previous mechanical loading, enabling faster responses to subsequent training and regenerative challenges. This review synthesizes current evidence on the cellular and epigenetic mechanisms underlying skeletal muscle memory and examines how these mechanisms are modified by aging and post-injury regeneration. Muscle memory emerges from complementary structural and molecular components, including myonuclear retention, persistent DNA methylation changes, chromatin remodeling, transcriptional priming, non-coding RNA regulation, and mitochondrial epigenetic adaptations. These mechanisms interact with muscle satellite cells (MuSCs), fibro-adipogenic progenitors (FAPs), immune cells, and extracellular matrix remodeling to maintain regenerative competence. During aging, epigenetic drift, chronic low-grade inflammation, altered macrophage states, MuSC dysfunction, persistent FAP activity, fibrosis, mitochondrial impairment, and anabolic resistance progressively reduce this plasticity, thereby contributing to sarcopenia. Training–detraining–retraining studies indicate that parts of the exercise-induced epigenetic landscape remain detectable after training cessation and can be reactivated during renewed loading, although the persistence and functional importance of individual molecular signatures remain incompletely defined. Physical exercise remains the most established intervention for preserving muscle function and epigenetic responsiveness, whereas caloric restriction, modulation of nutrient-sensing pathways, senolytic strategies, and direct targeting of epigenetic regulators remain promising but translationally less mature approaches. Overall, the preservation of epigenetic plasticity may be a key determinant of healthy skeletal muscle aging and effective regeneration.

## 1. Introduction

Physical exercise and caloric restriction are among the most effective non-pharmacological interventions known to delay the molecular manifestations of aging. Rather than acting through a single mechanism, both interventions induce extensive metabolic remodeling that coordinates nutrient sensing, mitochondrial adaptation, proteostasis, stress resistance, and epigenetic regulation, thereby influencing multiple hallmarks of biological aging. In skeletal muscle, these adaptive responses extend beyond systemic metabolic homeostasis and directly regulate the maintenance, regenerative potential, and long-term functional competence of muscle satellite cells (MuSCs). Experimental studies have demonstrated that even short-term caloric restriction significantly enhances the survival and regenerative capacity of MuSCs in murine models [1]. Mechanistically, these effects partially converge with signaling pathways targeted by several extensively studied geroprotective compounds, including resveratrol, rapamycin, and metformin. Although these interventions act through distinct primary molecular targets, they share the ability to modulate conserved nutrient-sensing pathways centered on AMP-activated protein kinase (AMPK), mechanistic target of rapamycin complex 1 (mTORC1), and sirtuin signaling, thereby promoting metabolic adaptation and cellular resilience [2,3,4,5,6]. However, the biological consequences of physical exercise extend well beyond these systemic metabolic adaptations. Skeletal muscle exhibits remarkably persistent epigenetic plasticity, in which prior hypertrophic stimuli leave durable molecular signatures that influence subsequent responses to mechanical loading. This phenomenon, commonly referred to as epigenetic muscle memory, is characterized by long-lasting alterations in DNA methylation and chromatin architecture that persist despite prolonged detraining, thereby priming skeletal muscle for more rapid and efficient adaptive remodeling upon renewed exercise [7]. Therefore, this review aims to synthesize current evidence on the cellular and epigenetic mechanisms underlying skeletal muscle memory, with particular emphasis on persistent molecular adaptations induced by physical activity and their modification during aging and post-injury regeneration. We further examine how age-related epigenetic drift, inflammatory remodeling, satellite cell dysfunction, and alterations in the regenerative niche interfere with these memory mechanisms, and discuss the potential of exercise and emerging epigenetic interventions to preserve regenerative competence in aging skeletal muscle.

## 2. Cellular Architecture and Tissue Compartments of Skeletal Muscle

### 2.1. Myofibers and Muscle Satellite Cells

Multinucleated muscle fibers (myofibers) are recognized as the primary contractile units of skeletal muscle tissue and are estimated to account for approximately between 35% to 40% of the total body mass of a healthy human [8,9]. They are composed of structures such as myofibrils and sarcomeres, and their unique architecture is formed through the fusion of multiple mononucleated muscle cells (myoblasts) [8,10]. These fibers are characterized as terminally differentiated, postmitotic cells and are therefore unable to re-enter the cell cycle or undergo further cell division [10]. Despite the lack of proliferative capacity, muscle fibers undergo hypertrophy and regeneration following injury, mediated by several mechanisms [8,9]. One of these mechanisms is associated with sarcomeres and involves the physical addition of new sarcomeres to the pre-existing myofibrillar structures within the muscle fiber [8]. This process, referred to as sarcomeric adjunction, occurs during hypertrophy, contributing to the physical enlargement of muscle cells by increasing both muscle fiber diameter and length [8,11]. During regeneration, the same process is involved in re-establishing the functional integrity of the contractile apparatus [9,12]. The second mechanism is closely linked to the myonuclear domain hypothesis, whereby each nucleus within a muscle fiber is considered to maintain genomic control over a specific volume of the surrounding cytoplasm [8,13]. The myonuclear domain hypothesis is closely associated with muscle fiber growth, as cytoplasmic volume expands during fiber hypertrophy, thereby increasing the demand for myonuclei. This demand is met by satellite cells [8]. MuSCs are recognized as specialized resident somatic stem cells of skeletal muscle tissue and are characterized by their ability to undergo both self-renewal and myogenic differentiation [10]. In the absence of the need for muscle repair or growth, MuSCs remain quiescent [8,12]. These cells are situated directly on the surface of the muscle fiber, between the sarcolemma and the surrounding basal lamina, a component of the extracellular matrix [12,14,15]. Their quantity varies by location, with the highest numbers at the ends of muscle fibers [12]. Upon activation by various stimuli, such as resistance training [13], postnatal growth [8], nitric oxide (released almost immediately following injury or in response to mechanical stretching) [9], sphingosine-1-phosphate [9], and other factors, satellite cells are induced to migrate. As the migratory capacity of these cells is greatest before intensive proliferation begins, movement along muscle fibers is observed prior to the onset of proliferation [9,12]. Upon reaching the target site, proliferation is initiated [9]. A proportion of these divisions is asymmetric, with one cell retaining stem cell identity while the other commits to the myogenic lineage and becomes a myoblast capable of contributing to muscle growth or regeneration, depending on the physiological requirements [8,9,12]. Ultimately, myocyte fusion is induced, leading either to the formation of new muscle fibers or to the incorporation of these cells into damaged areas of pre-existing muscle fibers, thereby enabling their repair [9,12].

### 2.2. Fibro-Adipogenic Progenitors and Extracellular Matrix Remodeling

Fibro-adipogenic progenitors (FAPs) are mesenchymal stromal cells residing within the interstitial connective tissue of skeletal muscle. Under physiological conditions, they remain largely quiescent, whereas following muscle injury, they rapidly expand and provide paracrine and structural support for regeneration. Rather than contributing directly to myofiber formation, FAPs regulate MuSC activity through trophic signaling and coordinate transient extracellular matrix (ECM) remodeling [10,15,16,17]. Their activation is influenced by signals derived from immune cells, including eosinophil-associated cytokines, and is followed by the production of a provisional ECM scaffold that supports regenerating myofibers.

Once regeneration progresses, this transient population is efficiently eliminated by programmed apoptosis, preventing the excessive accumulation of connective tissue. Maintenance of this tightly controlled program depends on retinoic acid signaling, which preserves FAPs in an immature state, together with a finely balanced interplay between the pro-apoptotic effects of tumor necrosis factor α (TNF-α) and the pro-survival signaling mediated by transforming growth factor β (TGF-β) and platelet-derived growth factor receptor alpha (PDGFRα) [18,19,20,21].

After activation, a rapid increase in the number of FAPs is observed, and the peak of their expansion occurs approximately 2–3 days after damage [16]. This process is accompanied by the production of trophic factors that influence immune system cells, and most importantly, MuSCs [10]. Among the factors influencing satellite cells are follistatin, Wingless-related integration site (Wnt1)-inducible signaling pathway protein-1 (WISP1), insulin-like growth factor 1 (IGF-1), and IL-6 [10,16,22]. The first of these factors, follistatin, is characterized as an antagonist of myostatin and is involved in promoting myoblast fusion, thereby recognized as a potent pro-myogenic factor [10]. The subsequent factors, WISP1 and IGF-1, are involved in regulating satellite cell expansion and proliferation [8,10]. Additionally, it is worth noting that an impaired increase in WISP1 expression by FAPs following muscle injury is observed in older individuals, a major factor contributing to age-related impairment of skeletal muscle regeneration [10,22,23]. The last-mentioned factor, IL-6, is involved in promoting the transition of cells from the proliferative stage to muscle fiber formation [10]. FAP-derived Wnt signals additionally influence MuSC fate. Canonical Wnt/β-catenin signaling contributes to myogenic differentiation, whereas non-canonical Wnt7a-dependent signaling supports planar cell polarity and symmetric expansion of the satellite-cell pool [12,24,25]. These pathways are discussed in greater detail in Section 3.4. Following acute muscle injury, FAPs rapidly expand and transiently deposit extracellular matrix components, including type I, III, and VI collagens and fibronectin, thereby creating a provisional scaffold that supports the alignment and regeneration of newly formed myofibers [8,23,26]. WISP1-positive FAP subpopulations are particularly enriched in matrix-remodeling factors and simultaneously provide trophic signals that support MuSC function [10,22]. The formation of such a scaffold aims to ensure the proper alignment of newly formed muscle fibers [10,23].

### 2.3. Immune Cells and the Inflammatory-to-Regenerative Transition

The immune response is rapidly activated following injury and is essential for regulating the transition from the initial pro-inflammatory phase to the subsequent regenerative phase [22]. Following injury, neutrophils are activated by damage-associated molecular patterns (DAMPs) and are rapidly recruited, with their presence being detected as early as 1–6 h post-injury [9,22]. Their activity is characterized by phagocytosing cellular debris and releasing pro-inflammatory cytokines, such as TNF-α and IL-1β [8,12,22]. In addition to pro-inflammatory cytokines, macrophage colony-stimulating factor (CSF1) and soluble interleukin-6 receptor (sIL-6R) are released, contributing to the recruitment of circulating monocytes and their subsequent differentiation into macrophages [8,22]. Approximately 24 h post-injury, the inflammatory response becomes dominated by M1 macrophages. TNF-α is secreted by these cells and promotes satellite cell proliferation, whereas during the later stages of the repair process (approximately 2 to 3 days post-injury), the same factor mediates the induction of apoptosis in excessive FAPs [8,12,23].

Skeletal muscle regeneration following injury is a highly coordinated process that depends on the interplay between muscle cells and the immune system. Although the reconstruction of damaged myofibers relies primarily on the activation, proliferation, and differentiation of MuSCs [27,28,29], effective tissue repair also requires a precisely regulated inflammatory response. Following injury, neutrophils, monocytes, and macrophages are recruited to the damaged tissue, where they remove necrotic cellular debris and actively regulate regenerative processes [30,31,32].

Macrophages represent the predominant immune cell population involved in the response to sterile muscle injury and play a pivotal role in determining the course and outcome of skeletal muscle regeneration. These cells exhibit considerable functional heterogeneity, originate from distinct developmental sources, and contribute both to tissue homeostasis and post-injury repair processes [33,34,35]. Under physiological conditions, resident macrophages continuously monitor the local muscle microenvironment and contribute to tissue homeostasis. Following injury, however, they become key orchestrators of both inflammatory and regenerative responses [36,37].

During the early phase of tissue damage, macrophages contribute to the initiation and organization of the inflammatory response by secreting chemotactic factors that recruit additional immune cells and by producing matrix metalloproteinases that facilitate cell migration into the injured tissue [38]. Macrophages also perform essential phagocytic functions by clearing necrotic myofibers, cellular debris, and apoptotic neutrophils, thereby creating a permissive environment for tissue repair. Impaired macrophage recruitment or activation results in the delayed clearance of damaged tissue, prolonged exposure to pro-inflammatory stimuli, and incomplete regeneration [36,37].

The inflammatory response following muscle injury proceeds sequentially and is tightly synchronized with the successive stages of myogenesis. During the early regenerative phase, pro-inflammatory macrophages, commonly referred to as M1 macrophages (cluster of differentiation 68-positive; CD68+), predominate. These cells phagocytose damaged myofibers and secrete pro-inflammatory cytokines, including tumor necrosis factor alpha (TNF-α) and interleukin-6 (IL-6), thereby promoting satellite cell proliferation [30]. Macrophages also support myoblast expansion through the secretion of granulocyte colony-stimulating factor (G-CSF), which acts on the transiently expressed granulocyte colony-stimulating factor receptor (G-CSFR) present on proliferating muscle progenitor cells [36].

Within the first hours after injury, neutrophils infiltrate the damaged tissue, followed by the accumulation of pro-inflammatory CD68+/(cluster of differentiation 163-negative) CD163− macrophages, which typically reach maximal abundance approximately 24 h after injury. These macrophages eliminate necrotic cellular debris through phagocytosis while secreting TNF-α and IL-1, thereby amplifying the initial inflammatory response. During the subsequent regenerative phase (approximately days 2–4), macrophages undergo phenotypic transition toward an anti-inflammatory (cluster of differentiation 68-negative) CD68−/(cluster of differentiation 163-positive) CD163+ state. Through the secretion of IL-10, these cells actively resolve inflammation while promoting MuSC proliferation and myogenic differentiation [12,39,40,41].

A critical stage of the regeneration process is the transition of macrophages from a pro-inflammatory to an anti-inflammatory state, referred to as macrophage polarization, typically observed between 2 and 3.5 days post-injury [22]. This transition is mediated by the stimulation of macrophages with IL-10, IL-4, and IL-13, resulting in the suppression of pro-inflammatory gene expression, including *TNF* (tumor necrosis factor α gene) and *CCL2* (C-C motif chemokine ligand 2 gene) [22]. These interleukins are primarily produced by FAPs and eosinophils [10,15]. The appearance of M2 macrophages is associated with the promotion of new tissue formation [8,12]. These cells are also involved in the secretion of growth factors, such as IGF-1, which promote satellite cell differentiation and fusion into muscle fibers [9]. Furthermore, extracellular matrix components are produced, and the survival of maturing myoblasts is supported by the prevention of apoptosis [8,9].

## 3. Pathophysiology of Muscle Dysfunction: Sarcopenia and Impaired Wound Healing

### 3.1. Desynchronization of the Inflammatory Phase and Inflammaging

Inflammaging refers to the chronic, low-grade, sterile inflammatory state that develops with advancing age, characterized by persistent activation of inflammatory signaling pathways and increased production of pro-inflammatory mediators. In skeletal muscle, this altered inflammatory environment disrupts the tightly coordinated sequence of immune-cell activation required for efficient regeneration. Muscle regeneration is driven by complex interactions among macrophages, MuSCs, regulatory T cells (Tregs), fibroblasts, endothelial cells, and FAPs. The coordinated activity of these cell populations is essential for maintaining the balance between inflammation, regeneration, and tissue remodeling. Although the M1/M2 nomenclature remains useful for describing broad functional tendencies during muscle regeneration, macrophage activation in vivo represents a dynamic continuum rather than a binary transition between two discrete phenotypes. Macrophages may simultaneously express markers associated with pro-inflammatory and reparative states, and their phenotype evolves in response to temporally changing signals within the regenerative niche. Accordingly, the terms M1 and M2 are used here as operational descriptors rather than as strictly defined cell populations. Efficient muscle repair, therefore, depends on the timely shift from predominantly pro-inflammatory toward increasingly reparative macrophage functions, whereas disruption of this dynamic transition contributes to persistent inflammation, excessive fibrosis, and impaired regeneration [36,38,42].

Prolonged persistence of M1 macrophages may impair muscle regeneration by maintaining a pro-inflammatory microenvironment and producing cytotoxic levels of nitric oxide (NO), thereby causing secondary myofiber damage and delaying the transition from the proliferative phase to myogenic differentiation [30,38].

A critical step in successful muscle repair is the phenotypic transition of macrophages toward an anti-inflammatory and pro-regenerative M2 phenotype (cluster of differentiation 163-positive and cluster of differentiation 206-positive; CD163+/CD206+). These macrophages suppress pro-inflammatory activity by secreting IL-10 and other anti-inflammatory mediators while simultaneously promoting myoblast differentiation, fusion, and the maturation of newly formed myofibers. Reparative macrophages also produce numerous growth factors, including IGF-1, platelet-derived growth factor (PDGF), and vascular endothelial growth factor (VEGF), which stimulate cell proliferation, angiogenesis, and tissue repair [38]. In addition, macrophage-derived TGF-β regulates fibroblast activity and extracellular matrix remodeling, thereby contributing to the structural organization of regenerating tissue [36,38,42].

The importance of macrophage phenotypic plasticity is also evident in chronic muscle diseases. In the mdx mouse model of Duchenne muscular dystrophy, the inflammatory infiltrate exhibits a more complex pattern than that observed following acute injury. Early infiltration by M1 macrophages is accompanied by the presence of M2a macrophages (interleukin-4 receptor-positive and cluster of differentiation 206-positive; IL-4R+/CD206+), which attenuate inflammatory cytotoxicity, whereas the subsequent accumulation of M2c macrophages is associated with the activation of regenerative mechanisms. These findings indicate that appropriate transitions between macrophage phenotypes are fundamental for effective muscle regeneration in both acute and chronic pathological conditions [30,36,42].

Given their involvement in all stages of tissue repair, from the initiation of inflammation and clearance of damaged structures to the promotion of regeneration and resolution of immune responses, macrophages are now recognized as key regulators of tissue homeostasis and repair. Their ability to dynamically adapt their phenotype and integrate signals from the local microenvironment is essential for successful regeneration. Dysregulation of macrophage activation or functional reprogramming may result in chronic inflammation, excessive fibrosis, and impaired muscle repair. Consequently, macrophages are increasingly regarded as promising therapeutic targets for enhancing skeletal muscle regeneration and restoring tissue function [36,38].

### 3.2. Satellite Cell Dysfunction and Inflammatory Remodeling of the Stem Cell Niche

The age-associated decline in skeletal muscle regenerative capacity cannot be attributed solely to cell-intrinsic dysfunction of MuSCs. Instead, it results from a complex interplay between progressive epigenetic alterations within MuSCs and profound remodeling of their surrounding stem cell niche. Consequently, the regenerative competence of aged MuSCs is determined not only by their intrinsic molecular state, but also by the biochemical and cellular composition of the local microenvironment. The pivotal contribution of the systemic environment to stem cell aging has been elegantly demonstrated by heterochronic parabiosis experiments, in which the exposure of aged animals to the circulation of young counterparts substantially restored satellite cell function and regenerative capacity [43]. These findings established that a considerable proportion of age-related regenerative decline is reversible and driven by extrinsic rather than irreversible intrinsic defects.

Aging progressively transforms the skeletal muscle niche into a chronically inflamed microenvironment that profoundly impairs tissue regeneration [44]. A central contributor to this process is the senescence-associated secretory phenotype (SASP), whose composition undergoes dynamic temporal remodeling during tissue repair. Rather than representing a static pro-inflammatory program, the SASP evolves through distinct functional states that are tightly regulated by neurogenic locus notch homolog protein 1 (*NOTCH1*) signaling. During the early phases of tissue repair, elevated *NOTCH1* activity suppresses the pro-inflammatory transcription factor CCAAT/enhancer-binding protein beta (C/EBPβ), thereby limiting inflammatory cytokine production while promoting TGF-β—and platelet-derived growth factor AA (PDGF-AA)-dependent tissue remodeling and transient fibrotic responses. As *NOTCH1* signaling subsequently declines, the secretory program shifts toward a predominantly inflammatory phenotype characterized by the increased secretion of IL-1β, IL-6, IL-8, and matrix-remodeling factors that facilitate immune cell recruitment and senescent cell clearance [45,46,47].

### 3.3. Inflammatory Signaling and JAK/STAT3-Dependent Regulation of Muscle Satellite Cells

Muscle satellite cells constitute the principal stem cell population responsible for skeletal muscle regeneration following exercise-induced stress and tissue injury [48,49]. Although aging is accompanied by progressive telomere shortening, accumulation of somatic mutations, and persistent DNA damage within MuSCs [50,51,52], their regenerative potential is determined to a large extent by epigenetic alterations that dictate how these cells interpret signals originating from the surrounding tissue niche [53,54,55].

Following acute muscle injury, tissue-resident mast cells and macrophages function as the earliest sensors of damage by rapidly releasing histamine, tryptase, and TNF-α, thereby initiating de novo synthesis of IL-6 and triggering the inflammatory cascade required for tissue repair [56,57].

The biological effects of this cytokine network are highly dependent on both temporal dynamics and microenvironmental context. During the acute regenerative phase, transient TNF-α exposure activates the pro-survival nuclear factor kappa B (NF-κB) and p38 mitogen-activated protein kinase (p38 MAPK) pathways in MuSCs, thereby facilitating their activation following injury [58]. Simultaneously, exercise-induced IL-6 released from contracting myofibers activates Janus kinase/signal transducer and activator of transcription 3 (JAK/STAT3) signaling in a paracrine manner, promoting transient expansion of myogenic progenitors required for efficient muscle regeneration and hypertrophic adaptation [59]. Beyond its local effects, acute IL-6 also contributes to systemic metabolic homeostasis by stimulating lipolysis, regulating glucose metabolism, and counterbalancing several pro-inflammatory actions of TNF-α [60].

In contrast, chronic exposure to SASP, characteristic of aging skeletal muscle, fundamentally alters cytokine signaling. Persistent activation of the JAK/STAT3 and NF-κB pathways drives loss of MuSC self-renewal capacity, promotes premature differentiation, and contributes to irreversible cell-cycle arrest or progressive myofiber atrophy [61,62,63]. Regenerative failure in aged skeletal muscle is further exacerbated by defective IL-33-dependent recruitment and the local accumulation of Tregs, thereby impairing the resolution of inflammation and efficient tissue repair [64].

### 3.4. Molecular and Epigenetic Regulation of Myogenesis

Successful skeletal muscle regeneration requires precise coordination of structural remodeling, intracellular signaling, and transcriptional reprogramming. Before MuSCs can re-enter the myogenic program, damaged myofibers must restore membrane integrity and reorganize their cytoskeletal architecture. These early events depend on tightly regulated calcium homeostasis and μ-calpain-mediated proteolysis, which facilitate controlled cytoskeletal remodeling. Under pathological conditions, such as muscular dystrophies, sustained calcium influx results in excessive calpain activation and uncontrolled degradation of structural proteins, thereby compromising muscle integrity and regenerative capacity [65,66,67].

Following activation, MuSC migration and spatial positioning within regenerating tissue are orchestrated by the coordinated expression of cluster of differentiation 34 (CD34) together with Eph receptor–ephrin signaling, ensuring efficient colonization of damaged regions while preserving tissue architecture [68,69].

Commitment to the myogenic lineage is governed by a hierarchical transcriptional network. Myogenic factor 5 (Myf5) and myoblast determination protein 1 (MyoD) initiate satellite cell activation and proliferative expansion, whereas myogenin promotes irreversible cell-cycle withdrawal and terminal differentiation through the formation of multinucleated myofibers. This transition is tightly coordinated with interactions between MyoD, cyclin-dependent kinase 4 (CDK4), and cyclin D1, thereby synchronizing cell-cycle progression with the initiation of differentiation [70,71].

Activation of the myogenic transcriptional program is further reinforced by epigenetic remodeling. AMPK-dependent phosphorylation of ten-eleven translocation methylcytosine dioxygenase 2 (*TET2*) promotes DNA demethylation at key myogenic loci, thereby increasing chromatin accessibility and facilitating the transcription of genes required for muscle regeneration [72,73]. Importantly, this mechanism highlights a pivotal link between cellular metabolism and chromatin state. During the early stages of regeneration, p38 MAPK phosphorylates MyoD, thereby regulating activation of the myogenin promoter and preventing premature differentiation through context-dependent transcriptional repression. More broadly, activation of the p38 MAPK pathway is indispensable for efficient exit from quiescence and entry of MuSCs into the cell cycle [74,75].

It is important to note that satellite cells retain paired box 7 protein (PAX7) expression during the proliferative phase; however, the expression of this marker is lost during the final stage of differentiation, when the cells are converted into myocytes [8,9]. Maintenance of the MuSC pool depends on pronounced functional heterogeneity. While Pax7+/Myf5+ cells preferentially commit to myogenic differentiation, the Pax7+/Myf5− subpopulation serves as the self-renewing stem cell reservoir responsible for long-term tissue maintenance. Persistent overexpression of Pax7 suppresses myogenin induction and delays terminal differentiation, thereby preserving stem cell identity [76,77]. Reversible quiescence of this reserve population is maintained by Sprouty1-mediated inhibition of fibroblast growth factor signaling [78]. However, aging progressively reduces both the number and functional competence of these progenitors, despite their retained multilineage differentiation potential encompassing myogenic, osteogenic, and adipogenic fates [79]. This decline is further exacerbated by age-associated accumulation of osteopontin, which markedly suppresses regenerative activity [80]. Additional regulation of the stem cell niche is provided by neuronal nitric oxide synthase (nNOS), whose localization is disrupted in Becker muscular dystrophy and altered during aging, as well as by systemic endocrine signals that collectively modulate regenerative efficiency [81,82,83,84].

A central regulatory event controlling MuSC fate is the dynamic transition between Notch and Wnt signaling. In quiescent satellite cells, Notch signaling, mediated by recombination signal binding protein for the immunoglobulin kappa J region (RBP-J) and microRNA-708 (miR-708), is crucial for maintaining stemness and preventing spontaneous differentiation. Genetic disruption of this pathway results in rapid depletion of the satellite cell pool [85,86,87,88].

As regeneration progresses, declining Notch activity is accompanied by increasing Wnt signaling, thereby driving commitment toward terminal myogenic differentiation. Whereas relatively low canonical Wnt activity supports proliferative expansion, stronger activation promotes differentiation and myofiber formation. In parallel, the non-canonical Wnt7a pathway establishes planar cell polarity and stimulates symmetric expansion of the MuSC pool, allowing tissue regeneration without exhausting the stem cell reservoir [24,25,89].

During aging, this tightly regulated signaling balance progressively deteriorates. Persistent activation of canonical Wnt signaling, together with elevated systemic TGF-β1, redirects muscle progenitors from a myogenic to a fibrogenic fate. Activation of β-catenin-dependent transcription induces the expression of myofibroblast-associated genes, including alpha-smooth muscle actin (α-SMA), resulting in excessive extracellular matrix deposition, progressive fibrosis, and the disruption of normal skeletal muscle architecture [90,91,92,93].

### 3.5. Fibrotic Remodeling of the Stem Cell Niche: Fibro-Adipogenic Progenitors and the Myostatin–Follistatin Axis

Transient fibrogenesis is not merely a by-product of tissue injury but represents a necessary component of successful regeneration. Pharmacological inhibition of this early fibrotic response, for example, using nilotinib, markedly compromises muscle repair, demonstrating that controlled extracellular matrix deposition is required before functional tissue reconstruction can occur. In parallel, type 2 immune signaling, particularly through IL-4, promotes physiological FAP activity, whereas the secretion of bone morphogenetic protein 3B (BMP3B) contributes to the preservation of mesenchymal homeostasis and protection against age-associated sarcopenia [15,94,95,96,97].

In the absence of FAP apoptosis and in an environment deprived of appropriate pro-regenerative signals, such as IL-4, differentiation of FAPs into adipocytes and fibroblasts is promoted [15,26].

The latter cell type is responsible for excessive collagen production, which leads to increased tissue stiffness and further stimulates collagen and TGF-β production by FAPs [23]. As a result, progressive scar formation is promoted despite the repair of the initial injury [23,26]. Ultimately, the mechanical properties of muscle tissue are compromised, and its regenerative capacity is progressively diminished [16,23]. Persistent FAP accumulation resulting from impaired apoptosis also negatively affects MuSC function [8,26]. The extracellular matrix becomes increasingly dense and rigid, which restricts the migration, differentiation, and fusion of satellite cells [23,26].

During aging and chronic muscle disease, this tightly regulated regenerative program progressively collapses. Instead of undergoing apoptosis, FAPs persist within the tissue, proliferate excessively, and increasingly differentiate into mature adipocytes and myofibroblasts, ultimately replacing functional contractile tissue with fibrotic extracellular matrix and ectopic fat deposits [26,98,99]. This pathological transition is regulated by several interconnected signaling pathways, including primary cilium-dependent signaling, the miR-22-3p/KLF6/MMP14 axis, and Wnt5a/GSK3/β-catenin signaling, all of which promote fibro-adipogenic differentiation under pathological conditions [100,101,102].

Under physiological conditions, canonical Wnt signaling, together with synergistic interactions between Notch and TNF-α, suppresses the excessive adipogenic expansion of FAPs, thereby preserving regenerative competence [103,104]. However, a chronic elevation of TNF-α within the aged or dystrophic niche progressively attenuates NOTCH1 activity, disrupting this regulatory network and favoring fibro-fatty degeneration [105]. Aging further impairs the regenerative secretome of FAPs by reducing the production of WISP1, an important activator of MuSC function, and interleukin-15 (IL-15), both of which contribute to the decline in regenerative capacity [106,107].

A central molecular regulator integrating these multicellular interactions is the myostatin–follistatin axis. Myostatin, a member of the TGF-β superfamily, acts as a negative regulator of skeletal muscle growth by suppressing myogenesis and promoting fibrotic remodeling [108,109]. The balance between myostatin and its endogenous antagonist, follistatin, provides an additional link among FAP activity, fibrosis, and myogenic regeneration. Follistatin is expressed at higher levels in FAPs than in MuSCs and locally antagonizes myostatin activity, thereby supporting a pro-myogenic environment during active regeneration [20,110]. Conversely, the persistence of FAPs and disruption of the myostatin–follistatin balance during aging or chronic injury may favor fibrotic remodeling and impair MuSC-mediated regeneration.

### 3.6. Cell-Autonomous Metabolic Aging of Muscle Satellite Cells and the Central Role of p38α/β MAPK

The age-related decline in skeletal muscle regeneration cannot be explained solely by the deterioration of the surrounding tissue environment. Elegant transplantation studies have demonstrated that aged MuSCs retain profound functional defects even after engraftment into a young regenerative niche, indicating that aging also induces stable, cell-autonomous alterations within the stem cells themselves [111,112].

One of the defining characteristics of aged MuSCs is progressive metabolic dysfunction. Compared with their youthful counterparts, senescent myogenic progenitors exhibit impaired glycolytic flux, reduced glucose uptake, and diminished metabolic flexibility, ultimately compromising their ability to rapidly activate the biosynthetic programs required for proliferation and tissue regeneration [113]. These metabolic abnormalities are accompanied by persistent oxidative stress, chronic inflammatory signaling, and progressive disruption of intracellular signaling networks that normally preserve stem cell quiescence and regenerative competence.

**Figure 1:**
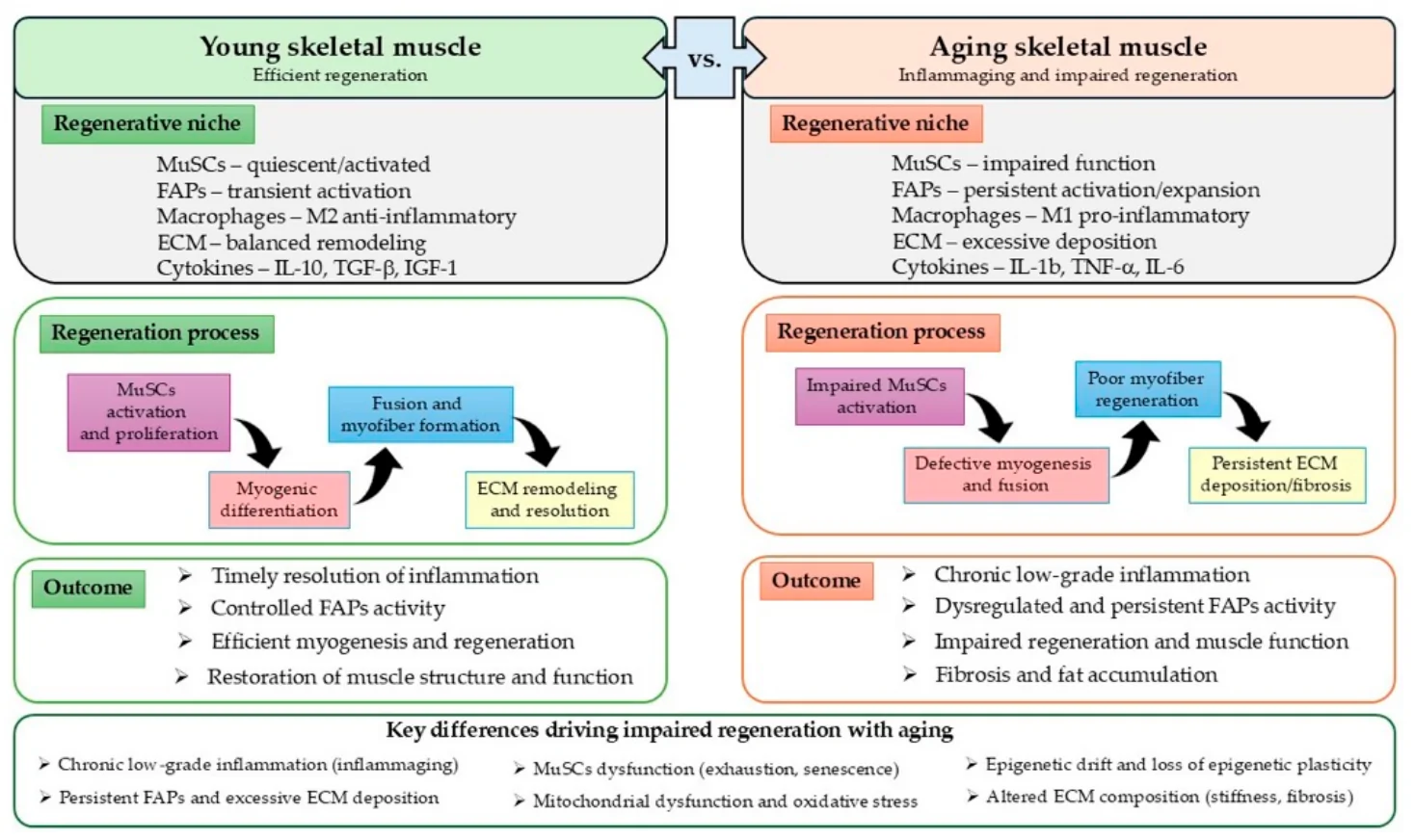
Age-related remodeling of the skeletal muscle regenerative niche. In young skeletal muscle, coordinated interactions among muscle satellite cells (MuSCs), fibro-adipogenic progenitors (FAPs), macrophages, cytokines, and the extracellular matrix (ECM) support efficient myogenesis, resolution of inflammation, and restoration of muscle structure and function. In aging skeletal muscle, chronic low-grade inflammation (inflammaging), persistent FAP activation, MuSC dysfunction, excessive ECM deposition, mitochondrial dysfunction, and epigenetic drift disrupt this coordinated regenerative response, promoting fibrosis, fat accumulation, and impaired muscle regeneration.

Among these pathways, the p38 MAPK family has emerged as one of the principal molecular determinants of MuSC aging. In young skeletal muscle, the p38α and p38β isoforms remain largely inactive during quiescence and are only transiently activated following injury to coordinate asymmetric cell division and commitment toward myogenic differentiation. This tightly regulated temporal activation enables efficient regeneration while simultaneously preserving the self-renewing stem cell pool. During aging, however, chronic exposure to inflammatory mediators associated with inflammaging, together with sustained oxidative stress, results in persistent hyperactivation of p38α/β signaling [112,114]. Rather than promoting physiological regeneration, constitutive p38 activity drives irreversible loss of quiescence, exhaustion of the stem cell compartment, and establishment of cellular senescence. Mechanistically, this process is associated with reduced activity of the transcription factor Slug (*SNAI2*), which, under physiological conditions, represses the expression of the cyclin-dependent kinase inhibitor p16INK4a. Loss of Slug-mediated repression therefore permits the accumulation of p16INK4a, reinforcing irreversible cell-cycle arrest and further limiting regenerative capacity [115]. The major age-related alterations in the skeletal muscle regenerative niche and their consequences for post-injury regeneration are summarized in Figure 1.

## 4. Molecular Basis of Skeletal Muscle Memory

Phenotypically, this molecular memory is reflected by the more rapid recovery of muscle mass and strength during retraining compared with the initial adaptive response [7,116]. Epigenetic skeletal muscle memory refers to the persistence of molecular changes induced by a previous physiological stimulus that modify the transcriptional response to subsequent loading. Among the mechanisms implicated in this process, DNA methylation, chromatin remodeling, histone modifications, transcriptional priming, and mitochondrial epigenetic changes can alter gene accessibility and responsiveness without modifying the underlying DNA sequence [7,117,118,119,120,121,122,123]. Importantly, in the context of muscle memory, the critical feature is not the presence of an epigenetic modification itself, but its persistence after the original training stimulus and its functional reactivation during subsequent retraining. All of these modifications are mediated by specific enzymes. DNA demethylation is mediated by enzymes such as TET1, whereas DNA methylation is catalyzed by DNA methyltransferases, including DNA methyltransferase 3A (DNMT3A), DNA methyltransferase 3B (DNMT3B), and methyltransferase 1 (DNMT1) [117].

New DNA methylation patterns (de novo) are established by DNMT3A and DNMT3B, for example, in response to environmental signals [120]. This process ensures mitotic stability and simultaneously serves as the foundation of DNA methylation memory [117,121].

Persistent changes in DNA methylation may provide a molecular substrate for transcriptional priming, whereby prior mechanical loading leaves selected genomic regions in a state that can be reactivated more rapidly upon subsequent exposure to the same stimulus. In skeletal muscle, this concept is supported by training–detraining–retraining studies demonstrating that a subset of exercise-induced hypomethylated loci does not return completely to baseline after training cessation. Training–detraining–retraining paradigms provide direct experimental evidence for persistent epigenetic memory in skeletal muscle. Following training, widespread DNA methylation remodeling is observed, with hypomethylation predominating at loci associated with skeletal muscle adaptation. Importantly, a subset of these loci remains hypomethylated during detraining and exhibits further hypomethylation or enhanced transcription during retraining, indicating that the epigenome does not fully return to its pre-training state [124]. Genome-wide DNA methylation analysis after high-intensity interval training (HIIT) demonstrated extensive remodeling of the skeletal muscle epigenome, characterized by a predominance of hypomethylation, indicating increased transcriptional accessibility. During detraining, the total number of differentially methylated positions (DMPs) decreased significantly but remained higher than the baseline. Hypomethylation continued to predominate, whereas the number of hypermethylated sites nearly returned to the pre-training levels. After retraining, the number of DMPs increased again, accompanied by a progressive increase in the proportion of hypomethylated loci. These findings suggest that the epigenome shifts toward a transcriptionally permissive state [124]. A gradual increase in the expression of *ADAM19*, *FOXK2*, *INPP5A*, *MTHFD1L*, and *THEM40* was observed. This elevated expression persisted throughout the detraining period and was maintained or further enhanced after retraining. An important mechanism underlying these changes was the stable hypomethylation of the promoters and regulatory regions of *ADAM19*, *INPP5A*, *MTHFD1L*, and *CAPN2*, which remained elevated regardless of whether training stimuli were present. These findings demonstrate that the epigenome is not fully reset after training ceases but instead remains in a state of increased transcriptional accessibility, providing a molecular basis for muscle memory [124].

As mentioned earlier, the increase in the number of cell nuclei is accompanied by elevated *EGR1* expression. It has been suggested that *EGR1* expression rises rapidly in previously untrained muscle but is transient. In contrast, in previously trained muscle, its induction during retraining occurs later but is sustained for a longer period [125].

Integrated methylome and transcriptome analyses have identified several genes that undergo training-induced epigenetic modifications and remain transcriptionally active after detraining, with their expression increasing further during retraining. These genes include *FLNB*, *UBR5*, *SETD3*, and *PLA2G16* [7]. Hypomethylation of selected CpG sites in skeletal muscle tissue persisted throughout the detraining period despite the loss of muscle mass. The genes *UBR5*, *RPL35A*, *HEG1*, *PLA2G16*, and *SETD3* exhibited the greatest increases in hypomethylation and gene expression during retraining, with expression levels exceeding those observed during the initial training period. Furthermore, the genes *GRIK2*, *TRAF1*, *BICC1*, and *STAG1* were identified as being epigenetically responsive to a single bout of intense resistance exercise. These genes became hypomethylated after a single resistance exercise session, with a hypomethylated profile also observed during subsequent loading and reloading. Notably, *GRIK2* and *TRAF1*, together with *AXIN1* and *CAMK4*, showed hypomethylation following loading that was retained during unloading and subsequent reloading, supporting their potential contribution to the persistent epigenetic component of muscle memory [7]. Taken together, the available human studies do not yet support a single universally replicated gene marker of skeletal muscle memory. Rather, the strongest evidence points to a recurring molecular pattern characterized by retained or recurrent DNA hypomethylation during detraining and/or retraining, accompanied by persistent or enhanced gene expression. Within the resistance-training model, *UBR5*, *RPL35A*, *SETD3*, *PLA2G16*, *AXIN1*, *GRIK2*, *CAMK4*, and *TRAF1* represent some of the best-characterized candidate genes, whereas HIIT has been associated with a partly distinct epigenetic memory signature involving *ADAM19*, *INPP5A*, *MTHFD1L*, *CAPN2*, and *SLC16A3*, suggesting that gene-level signatures of skeletal muscle memory may be partly exercise-modality specific. Thus, although the specific molecular signatures vary between exercise modalities, both resistance training and HIIT provide evidence that exercise-induced epigenetic modifications can persist or re-emerge after detraining [7,124]. Whether aging alters the establishment or persistence of these molecular muscle-memory signatures remains to be fully determined [126,127].

Beyond these epigenetic signatures, the concept of skeletal muscle memory has evolved to encompass multiple complementary mechanisms. Rather than representing a single biological phenomenon, current evidence indicates that long-term adaptation to mechanical loading arises from the integration of two complementary forms of cellular memory: a relatively stable structural component and a dynamic molecular program encoded through epigenetic and transcriptional regulation. Together, these mechanisms enable skeletal muscles to respond more rapidly and efficiently to repeated physiological stimuli while simultaneously preserving regenerative competence. Gene expression is regulated not only through DNA modifications, but also through a variety of post-translational modifications of histone proteins. Similar to DNA modifications, histone modifications are mediated through the dynamic addition or removal of specific chemical groups [123]. Among the best-characterized post-translational histone modifications are methylation, acetylation, phosphorylation, and ubiquitination.

During histone methylation, methyl groups are covalently attached to lysine (K) or arginine (R) residues, primarily within histone H3, and, depending on the residue and position modified, either chromatin relaxation or condensation may be induced, thereby influencing gene expression [119,123]. Methylation of histone residues, such as H3K9 and H3K27, promotes heterochromatin formation and contributes to gene silencing [123]. Conversely, H3K4 methylation is linked to chromatin relaxation and the active transcriptional state of genes [123].

**Figure 2:**
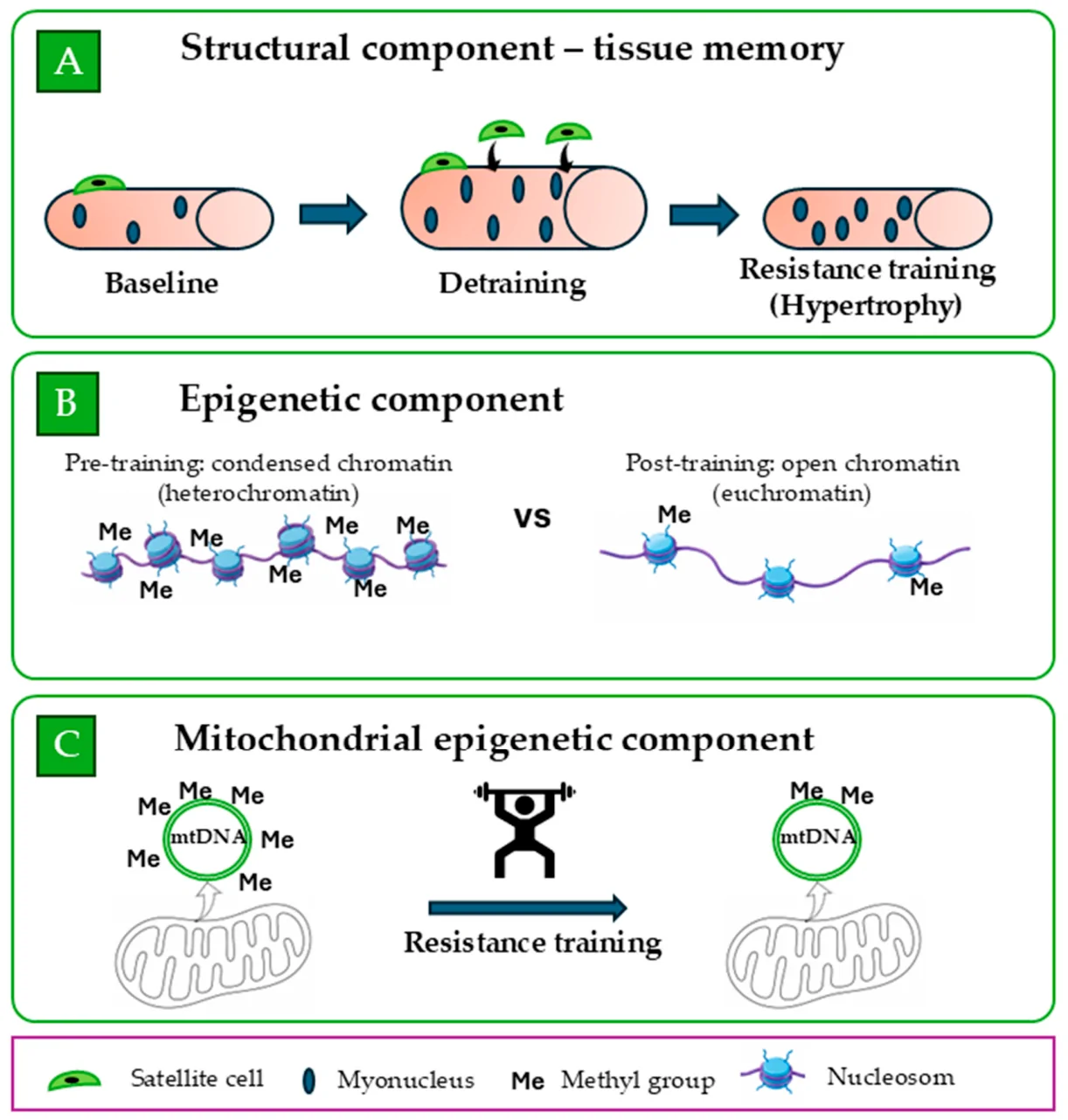
A multilevel model of integrated muscle memory in response to resistance training, detraining, and the aging process. (**A**) Structural component of tissue memory: Mechanical loading of skeletal muscle induces the activation of satellite cells, leading to their proliferation and fusion with existing muscle fibers. The newly added myonuclei precede muscle hypertrophy and exhibit remarkable stability—they are permanently retained within the muscle fiber syncytium and are not lost during subsequent periods of detraining or acute muscle fiber atrophy in young individuals [128,129]. This memory displays strict compartmental specificity: while myonuclei are responsible for maintaining the structural integrity of muscle fibers, the nuclei of interstitial connective tissue niche cells (including fibro-adipogenic progenitors, FAPs) permanently retain a transcriptional readiness for renewed mechanical loading [130]. (**B**) Epigenetic component: The imprint of previous training (hypertrophic) cycles is preserved at the level of nuclear cellular programming. The initial phase of mechanical loading induces widespread removal of methyl groups (hypomethylation) from thousands of genes. During detraining, despite a reduction in muscle fiber size, the DNA methylation profile does not fully return to its baseline state, maintaining an open chromatin configuration (euchromatin) that facilitates more rapid re-expression of genes upon retraining. This phenomenon involves key regulators of muscle growth and differentiation, including AXIN1 and AXIN2 (components of the Wnt/β-catenin signaling pathway) and elements of the anabolic PI3K/Akt/mTOR signaling cascade [7]. (**C**) Mitochondrial epigenetic component: An integral, autonomous component of the adaptive response in skeletal muscle consists of changes occurring directly within the mitochondrial genome (mtDNA). Resistance training has been shown to reverse age-related epigenetic alterations in mitochondria by inducing extensive hypomethylation affecting up to 63% of the examined CpG sites across the mitochondrial genome. This effect of epigenetic rejuvenation is particularly pronounced within the regulatory D-loop region, relieving transcriptional repression and directly enhancing the expression of subunits of respiratory chain complexes III and IV, thereby restoring a more youthful bioenergetic capacity to mitochondria in older individuals [131].

Histone modifications provide an additional layer of transcriptional regulation that may contribute to skeletal muscle memory by altering chromatin accessibility and the responsiveness of exercise-regulated genes. Active chromatin states are generally associated with histone acetylation and permissive marks such as H3K4 methylation, whereas repressive modifications, including H3K27me3, restrict transcriptional accessibility [121,122,123]. However, compared with DNA methylation, direct evidence for the persistence of specific histone modifications throughout training–detraining–retraining cycles in human skeletal muscle remains limited. Therefore, histone remodeling should currently be considered a plausible, complementary mechanism underlying muscle memory rather than an independently established, persistent molecular signature. The multilevel mechanisms contributing to skeletal muscle memory are summarized in Figure 2.

## 5. Epigenetic Mechanisms Underlying the Loss of Regenerative Capacity

### 5.1. Age-Associated Epigenetic Drift

One of the key causes of the decline in the regenerative capacity of aging muscle tissue is progressive epigenetic drift. This term describes the age-dependent, gradual loss of stability in gene expression regulatory patterns, including changes in DNA methylation, post-translational histone modifications, chromatin reorganization, and disruptions in the function of non-coding RNAs [132,133,134]. In young muscle tissue, genes responsible for hypertrophy, protein synthesis, and satellite cell activation exhibit high epigenetic plasticity, enabling the dynamic switching of chromatin states between euchromatin and heterochromatin [134,135,136]. This applies, among others, to genes such as the *IGF-1/MGF* axis, the androgen receptor (AR), components of the mTORC1 pathway (e.g., MTOR, RPTOR), and regulators of ribosome biogenesis. This open and plastic chromatin organization enables rapid activation of the anabolic response to mechanical and metabolic stimuli [137,138,139,140,141]. However, with age, global chromatin reorganization occurs, and the homeostatic balance between euchromatin and heterochromatin is lost [132,142]. This process involves increased DNA methylation in regulatory regions and an increased proportion of repressive histone modifications such as H3K27me3, accompanied by a simultaneous decrease in markers of active transcription [142,143,144]. These global disruptions in genomic architecture have a particularly detrimental effect on specific genomic loci controlling muscle tissue homeostasis. These changes demonstrate tissue- and cell-specificity, affecting key promoters and enhancers in both satellite cell progenitors and mature muscle fibers [133,134,143]. In MuSCs, hypermethylation of CpG islands within genes that govern myogenesis (e.g., *MYOD1*) impairs the tissue’s regenerative potential. In postmitotic, mature muscle fibers, similar changes affect genes responsible for protein synthesis and mechanical signal transduction. The consequence of this phenomenon is a weakening of chromatin remodeling mechanisms, leading to a functional disconnection between the environmental signal and the cellular genomic response. Even in the presence of receptor stimulation or amino acid availability, this signal encounters an epigenetic barrier. Limited inducibility of effector genes, including IGF-1 and regulators of ribosomal protein synthesis, directly perpetuates the anabolic resistance phenotype [138,139,145]. This phenomenon constitutes an additional, autonomous level of molecular blockade that overlaps with deficits in cellular signaling cascades, forming one of the fundamental pillars of sarcopenia [133,142]. These age-associated alterations progressively reduce skeletal muscle’s ability to maintain and reactivate adaptive transcriptional programs, thereby providing a mechanistic link among epigenetic drift, anabolic resistance, and the erosion of previously acquired molecular adaptations.

### 5.2. Age-Related Erosion of Skeletal Muscle Memory

Following repeated periods of limb immobilization, the promoters of anabolic genes, enhancers of acetylcholine receptor (AChR) genes, and regions of mitochondrial DNA remain hypomethylated, with some methylation changes becoming permanent. After the first episode of muscle atrophy induced by immobilization, genes involved in muscle protein degradation and structural remodeling become activated. At the same time, the expression of genes responsible for protein synthesis and normal mitochondrial function is reduced [146]. The persistence of selected epigenetic modifications during periods of reduced mechanical loading indicates that disuse does not necessarily erase previously acquired molecular adaptations. However, aging may compromise the functional expression of this memory by reducing chromatin plasticity, increasing epigenetic drift, and attenuating the transcriptional response to renewed loading. Thus, retained epigenetic marks may coexist with a progressively impaired capacity to translate molecular memory into an effective adaptive response.

The structural component of muscle memory is primarily attributed to myonuclear retention. During hypertrophic growth, activation of MuSCs leads to fusion with existing myofibers and the incorporation of newly acquired myonuclei into the multinucleated syncytium. Classical studies have demonstrated that these myonuclei exhibit remarkable long-term stability and frequently persist despite prolonged muscle unloading or subsequent atrophy, thereby providing an expanded transcriptional capacity that facilitates accelerated re-hypertrophy following renewed mechanical loading [116,128,147].

The extent of myonuclear accretion depends on the magnitude of hypertrophic adaptation. Relatively modest increases in muscle size are accompanied by limited myonuclear addition, whereas more pronounced hypertrophy induces substantially greater incorporation of new nuclei. Importantly, elevated myonuclear density has also been documented in former users of anabolic-androgenic steroids, suggesting that structural adaptations acquired during previous anabolic stimulation may persist for many years [148,149,150]. Similarly, resistance training in older adults increases both satellite cell abundance and myonuclear number while largely preserving the myonuclear domain despite age-associated fluctuations in muscle fiber size [151,152,153].

Nevertheless, the long-term persistence of myonuclei remains an area of active debate. Studies performed in rapidly adapting fast-twitch muscles, particularly the plantaris, have reported partial loss of myonuclei during prolonged detraining, suggesting that structural muscle memory is not universally permanent [116,154]. Evidence further indicates that newly acquired myonuclei generated during hypertrophy may display selective susceptibility to apoptosis under specific physiological conditions [155]. This process appears to be influenced by both the cellular age and integrity of the neuromuscular junction. During sarcopenia, impaired p38 MAPK signaling disrupts the agrin–MuSK axis, promoting denervation and selective elimination of specialized perisynaptic myonuclei through nucleophagy, ultimately compromising synaptic maintenance and muscle function [156].

Importantly, even when components of this structural “hardware” are partially lost, skeletal muscle retains a second, more dynamic layer of biological memory encoded by persistent epigenetic and transcriptional adaptations [154].

In addition to the DNA methylation patterns described above, the molecular component of skeletal muscle memory involves coordinated histone modifications, regulation of peroxisome proliferator-activated receptor gamma coactivator 1-alpha (PGC-1α) methylation by DNMT3B and TET1, and the AMPK/SIRT1/class IIa histone deacetylase (HDAC) signaling axis, which collectively regulate mitochondrial biogenesis, Glucose Transporter Type 4 (GLUT4) expression, lipid oxidation, and metabolic adaptation in response to exercise and disuse [157,158,159,160,161,162,163,164]. Although endurance training also improves inflammatory status in older individuals, its ability to remodel this epigenetic landscape appears to be attenuated with advancing age [165].

Beyond epigenetic modifications, muscle memory also encompasses a persistent transcriptomic component, involving messenger RNAs, non-coding RNAs, and microRNAs, which maintains MuSCs in a transcriptionally primed “alert” state. Single-nucleus analyses further demonstrate that this memory is highly compartmentalized. Whereas myonuclei primarily retain metabolic adaptations, interstitial cell populations, including FAPs, preserve distinct DNA hypomethylation signatures that enable the rapid reactivation of secretory programs during subsequent regenerative challenges [130].

### 5.3. Mitochondrial and Stem-Cell Epigenetic Dysfunction

Mitochondrial epigenetic dysregulation represents an additional component of the age-related decline in skeletal muscle plasticity. Importantly, some of these alterations appear to remain responsive to exercise. In older skeletal muscle, six weeks of resistance training induced extensive hypomethylation of mitochondrial DNA, with the most pronounced changes occurring within the regulatory D-loop region. This mitochondrial epigenetic remodeling was associated with enhanced mitochondrial gene transcription, increased abundance of oxidative phosphorylation complex III and IV proteins, and a methylation profile that more closely resembled that observed in young muscle. These findings suggest that exercise may partially rejuvenate the mitochondrial epigenome, providing an additional mechanism through which epigenetic muscle memory contributes to the preservation of mitochondrial function during aging [131]. Collectively, these observations indicate that the relevance of muscle memory to aging extends beyond the response to retraining. The persistence of structural and molecular adaptations may help maintain skeletal muscle adaptive capacity, whereas age-related alterations in these mechanisms may progressively limit the muscle’s ability to respond to subsequent physiological stimuli.

Aging progressively erodes both structural and molecular components of this integrated memory system. Single-cell transcriptomic analyses reveal that aged MuSCs undergo extensive DNA methylation drift, resulting in increased transcriptional heterogeneity between individual cells [166]. This stochastic epigenetic drift disrupts coordinated gene expression programs, promotes premature activation of differentiation pathways at the expense of stemness, and ultimately compromises the ability of MuSCs to correctly interpret regenerative cues from the surrounding tissue niche. Consequently, loss of epigenetic fidelity emerges as a fundamental molecular mechanism linking aging to impaired muscle regeneration and progressive sarcopenia. The major age-associated epigenetic alterations affecting skeletal muscle plasticity, along with potential strategies to counter these changes, are summarized in Table 1.

## 6. Future Therapeutic Perspectives: Targeting the Epigenome to Preserve Skeletal Muscle Function

Recognition of epigenetic remodeling as a central regulator of skeletal muscle adaptation has shifted therapeutic strategies toward preserving epigenetic plasticity. Future interventions will likely focus on maintaining chromatin accessibility, supporting stem cell function, and limiting age-related epigenetic drift to delay sarcopenia and enhance regeneration. Given the multifactorial nature of muscle aging, effective therapies will probably combine lifestyle interventions with pharmacological approaches targeting nutrient sensing, inflammation, cellular senescence, and chromatin remodeling. However, the translational maturity of these approaches varies considerably, and their clinical application is limited by differences between experimental models and human aging, interindividual heterogeneity, tissue specificity, and uncertainty regarding the optimal timing and duration of the intervention.

### 6.1. Physical Exercise as an Epigenetic Intervention

Regular physical exercise remains the most effective strategy for preserving muscle function. It induces broad epigenetic adaptations that support mitochondrial function, metabolic regulation, and regenerative capacity while improving the muscle microenvironment. Exercise should therefore be considered a key physiological regulator of the muscle epigenome. A clinically relevant extension of exercise-induced muscle plasticity is prehabilitation, particularly resistance training performed before planned orthopedic surgery. Such interventions may improve muscle mass, strength, and neuromuscular function, and potentially induce molecular adaptations that enhance regenerative readiness. However, direct evidence linking prehabilitation-induced epigenetic remodeling to faster postoperative recovery, fewer complications, or improved functional outcomes remains limited, and these potential benefits require further validation in longitudinal clinical studies. Nevertheless, exercise-induced molecular and functional adaptations show substantial interindividual variability and are influenced by age, frailty, baseline training status, exercise modality, intensity, duration, and adherence. Therefore, defining exercise protocols that induce durable epigenetic adaptations while remaining feasible and safe for older or frail individuals remains an important translational challenge.

### 6.2. Caloric Restriction and Metabolic Reprogramming

Caloric restriction and related metabolic interventions may support muscle regeneration by modulating nutrient-sensing pathways and influencing chromatin organization through metabolite availability. These mechanisms have inspired the development of caloric restriction mimetics as potential therapeutic tools. However, prolonged caloric restriction may be difficult to implement in older individuals, and if inadequately controlled, may exacerbate loss of lean mass and compromise nutritional status. Consequently, its potential benefits for epigenetic and metabolic regulation must be balanced against the risk of accelerating sarcopenic muscle loss, particularly in frail older adults.

### 6.3. Pharmacological Modulation of Nutrient-Sensing Pathways

Compounds such as metformin, rapamycin, and resveratrol show promise by targeting conserved metabolic pathways that also influence epigenetic regulation. However, modulation of nutrient-sensing pathways may also interfere with physiological anabolic responses to exercise, and the consequences are likely to depend on compound, dose, treatment duration, metabolic status, and timing relative to exercise. Establishing regimens that preserve potential geroprotective effects without compromising muscle protein synthesis or regenerative adaptation will therefore be essential for long-term clinical application.

### 6.4. Epigenetic Therapies

Direct targeting of epigenetic enzymes, including HDACs, DNMTs, and TET proteins, represents a promising but still developing area. A major limitation of this approach is the broad biological activity of these enzymes, which regulate transcription in multiple tissues and cell types. Systemic modulation may therefore induce widespread epigenetic changes beyond skeletal muscle, with potentially unpredictable long-term consequences. Future strategies will require greater tissue and cell-type specificity, particularly if the aim is to selectively restore adaptive epigenetic states in myofibers, MuSCs, or other components of the regenerative niche.

### 6.5. Senolytic Therapies and Rejuvenation of the Regenerative Niche

Elimination or modulation of senescent cells may improve muscle regeneration by reducing chronic inflammation and restoring tissue homeostasis. However, senescent cells are heterogeneous and may exert context-dependent functions during tissue remodeling and repair. The therapeutic benefit of senolytic strategies will therefore depend on appropriate target-cell selection, timing, and dosing, while long-term safety and the consequences of eliminating specific senescent cell populations require further investigation.

### 6.6. Targeting Fibro-Adipogenic Progenitors and Fibrosis

Therapies aimed at regulating fibro-adipogenic progenitors and limiting fibrosis may enhance regeneration. Modulation of pathways such as myostatin signaling highlights the importance of restoring the broader regenerative environment rather than focusing solely on muscle mass. Importantly, FAPs are not exclusively pathological cells but are required for normal regeneration, where they provide trophic support and contribute to transient ECM remodeling. Therapeutic strategies should therefore aim to prevent persistent or maladaptive FAP activity rather than broadly suppress this population, as excessive inhibition could itself compromise tissue repair.

### 6.7. Precision Medicine and Future Directions

Advances in single-cell and multi-omics technologies are paving the way for personalized therapeutic strategies. Future interventions will likely target specific cellular and molecular defects within the muscle regenerative network to restore overall regenerative competence rather than simply increasing muscle mass. Partial epigenetic reprogramming has emerged as a potential strategy for restoring selected molecular features of youthful tissue function; however, its applicability to skeletal muscle remains largely experimental, and major questions regarding safety, durability, cell specificity, and preservation of cellular identity remain unresolved.

### 6.8. Translational Challenges

Despite rapid progress in understanding the molecular mechanisms underlying skeletal muscle aging and epigenetic memory, translation of these findings into clinical interventions remains challenging. Much of the mechanistic evidence derives from animal models, cultured cells, or relatively small human studies, and the extent to which these findings reflect long-term adaptation in aging human skeletal muscle remains uncertain. Aging itself is highly heterogeneous, with differences in biological age, frailty, physical activity, comorbidities, nutritional status, and medication use potentially modifying both baseline epigenetic states and responses to intervention. Moreover, epigenetic regulation is highly tissue- and cell-specific, making it difficult to selectively target myofibers, MuSCs, FAPs, or immune-cell populations without inducing systemic effects. The optimal timing, duration, and reversibility of epigenetic interventions also remain poorly defined. Finally, no validated epigenetic biomarker currently allows the reliable prediction of regenerative capacity or therapeutic response in individual patients. Longitudinal human studies integrating epigenomic and transcriptomic profiling with clinically relevant outcomes, including muscle strength, mobility, recovery after injury, and functional independence, will therefore be essential for translating experimental findings into personalized interventions.

## 7. Conclusions

Skeletal muscle adaptation results from the coordinated interaction of multiple cellular populations, signaling pathways, and epigenetic regulatory mechanisms that collectively preserve tissue integrity throughout life. Although mature myofibers constitute the principal contractile component of skeletal muscle, efficient regeneration depends equally upon satellite cells, fibro-adipogenic progenitors, immune cells, vascular elements, and extracellular matrix remodeling. These components function as an integrated regenerative network rather than as independent biological systems.

A central theme emerging from recent research is that epigenetic regulation constitutes the molecular interface linking environmental stimuli with long-term skeletal muscle adaptation. DNA methylation, histone modifications, chromatin remodeling, and non-coding RNAs dynamically regulate transcriptional accessibility, allowing skeletal muscle to adapt to mechanical loading, metabolic stress, and tissue injury. Repeated exercise establishes persistent epigenetic signatures that facilitate more rapid and efficient adaptive responses during subsequent mechanical stimulation, providing the molecular foundation of skeletal muscle memory.

The contemporary concept of muscle memory, therefore, extends beyond the classical myonuclear hypothesis. While the retention of myonuclei contributes to the preservation of transcriptional capacity, durable epigenetic remodeling provides an additional, highly dynamic layer of biological memory that enhances future regenerative and hypertrophic responses. Structural and molecular memory should therefore be regarded as complementary rather than mutually exclusive mechanisms.

Aging progressively erodes this adaptive capacity through the accumulation of epigenetic drift, chronic inflammation, satellite cell dysfunction, fibro-adipogenic progenitor persistence, extracellular matrix fibrosis, and anabolic resistance. Rather than reflecting the failure of isolated signaling pathways, age-associated regenerative decline represents a disruption of an integrated multicellular network whose function depends on preserved chromatin plasticity and coordinated intercellular communication.

Importantly, these age-associated alterations are not entirely irreversible. Regular physical exercise remains the most effective intervention capable of maintaining epigenetic plasticity, preserving satellite cell function, improving mitochondrial health, and limiting chronic inflammatory remodeling. Emerging pharmacological strategies targeting nutrient-sensing pathways, chromatin-modifying enzymes, senescent cells, and the regenerative niche may further complement the beneficial effects of exercise and offer novel therapeutic opportunities to prevent sarcopenia.

Future studies integrating single-cell epigenomics, spatial transcriptomics, and functional genomics will continue to refine our understanding of skeletal muscle adaptation. Such advances are expected to facilitate the development of precision therapies aimed not merely at increasing muscle mass, but at restoring the regenerative competence of aging skeletal muscle. Ultimately, preservation of epigenetic plasticity is one of the central determinants of healthy muscular aging and lifelong functional independence.

## Figures and Tables

**Table 1:** Epigenome modulation as a strategy to preserve skeletal muscle function.

Context	Mechanism	Examples/Molecular Features	Functional Consequence	Evidence Cited in This Review
Training/retraining	Persistent DNA hypomethylation	*ADAM19*, *INPP5A*, *MTHFD1L*, *CAPN2*; *UBR5*, *SETD3*, *PLA2G16* and other candidate loci	Transcriptional priming and enhanced responsiveness to renewed loading	[7,124]
Training/detraining	Structural memory	Myonuclear accretion and partial retention after unloading	Preserved transcriptional capacity during reloading	[116,128,147]
Exercise in older muscle	Mitochondrial epigenetic remodeling	mtDNA hypomethylation, including the D-loop; OXPHOS complex III/IV changes	Improved mitochondrial transcriptional competence	[131]
Aging	Epigenetic drift	Altered DNA methylation, repressive chromatin states, increased cell-to-cell variability	Reduced anabolic and regenerative responsiveness	[132,133,134,135,136,137,138,139,140,141,142,143,144,166,167]
Acute injury	Metabolism–epigenome coupling	AMPK/TET2, p38 MAPK, MyoD-related chromatin regulation	Activation and differentiation of MuSCs	[72,73,74,75]
Post-injury regeneration	Niche-dependent epigenetic regulation	MuSCs, FAPs, and immune-cell signaling; SASP and inflammatory pathways	Coordination of proliferation, differentiation, and ECM remodeling	[22,43,44,45,46,47,53,54,55,56,57,58,59,60,61,62,63,64]
Chronic aging/disease	Fibro-adipogenic remodeling	Persistent FAPs, Wnt/Notch/TGF-β signaling, myostatin–follistatin imbalance	Fibrosis, ectopic fat, and impaired MuSCs function	[26,96,99,100,101,102,103,104,105,106,107,108,109,110]

Abbreviations: AMP-activated protein kinase (AMPK), extracellular matrix (ECM), fibro-adipogenic progenitors (FAPs), p38 mitogen-activated protein kinase (p38 MAPK), mitochondrial genome (mtDNA), muscle satellite cells (MuSCs), oxidative phosphorylation (OXPHOS), senescence-associated secretory phenotype (SASP), translocation methylcytosine dioxygenase 2 (TET2).

## Data Availability

No new data were created or analyzed in this study. Data sharing is not applicable to this article.

## Abbreviations

The following abbreviations are used in this manuscript:

5mC	5-Methylcytosine
AChR	Acetylcholine receptor
AR	Androgen receptor
AMPK	AMP-activated protein kinase
BMP3B	Bone morphogenetic protein 3B
*CCL2*	C-C motif chemokine ligand 2 gene
CD68–	Cluster of differentiation 68-negative
CD68+	Cluster of differentiation 68-positive
CD163–	Cluster of differentiation 163-negative
CD163+	Cluster of differentiation 163-positive
CD206+	Cluster of differentiation 206-positive
CDK4	Cyclin-dependent kinase 4
CSF1	Macrophage colony-stimulating factor
DAMPs	Damage-associated molecular patterns
DMD	Duchenne muscular dystrophy
DMPs	Differentially methylated positions
DNMT	DNA methyltransferase
ECM	Extracellular matrix
FAPs	Fibro-adipogenic progenitors
G-CSF	Granulocyte colony-stimulating factor
G-CSFR	Granulocyte colony-stimulating factor receptor
GLUT4	Glucose Transporter Type 4
GRIK2	Glutamate receptor signaling
HATs	Histone acetyltransferases
HDACs	Histone deacetylases
HIIT	High-intensity interval training
IGF-1	Insulin-like growth factor 1
IL	Interleukin
IL-4R	interleukin-4 receptor-positive
JAK/STAT3	Janus kinase/signal transducer and activator of transcription 3
K	Lysine
MeCP2	Methyl-CpG Binding Protein 2
mTORC1	Mechanistic target of rapamycin complex 1
miR-708	microRNA-708
MSK1	Mitogen- and stress-activated kinase 1
mtDNA	Mitochondrial genome
MuSCs	Muscle satellite cells
Myf5	Myogenic factor 5
MyoD	Myoblast determination protein 1
*MYOD1*	Myogenic Differentiation 1 gene
nNOS	Neuronal nitric oxide synthase
NO	Nitric oxide
NOTCH1	Neurogenic locus notch homolog protein 1
NF-κB	Nuclear factor kappa B
p38 MAPK	p38 mitogen-activated protein kinase
PAX7	Paired box 7 protein
PCP	Planar cell polarity
PDGF	Platelet-derived growth factor
PDGFRα	Platelet-derived growth factor receptor alpha
PGC-1α	Peroxisome proliferator-activated receptor gamma coactivator 1-alpha
RBP-J	Recombination signal binding protein for immunoglobulin kappa J region
S	Serine
SASP	Senescence-associated secretory phenotype
sIL-6R	Soluble interleukin-6 receptor
*SNAI2*	Transcription factor Slug gene
TET2	Translocation methylcytosine dioxygenase 2
*TET2*	Tet methylcytosine dioxygenase 2 gene
TGF-β	Transforming growth factor β
TNF-α	Tumor necrosis factor α
*TNF*	Tumor necrosis factor α gene
Tregs	Regulatory T cells
VEGF	Vascular endothelial growth factor
WISP1	Wnt1-inducible signaling pathway protein-1
Wnt	Wingless-related integration site
